# Benchmarking halcyon ring delivery system for hypofractionated breast radiotherapy: Validation and clinical implementation of the fast‐forward trial

**DOI:** 10.1002/acm2.14047

**Published:** 2023-05-23

**Authors:** Damodar Pokhrel, Mason Smith, Alexander Volk, Mark E. Bernard

**Affiliations:** ^1^ Department of Radiation Medicine University of Kentucky Lexington Kentucky USA

**Keywords:** AcurosXB algorithm, APBI‐VMAT, Co‐planar geometry, Fast‐Forward trial, Halcyon RDS

## Abstract

**Purpose:**

The aim of this study was to demonstrate the feasibility and efficacy of an iterative CBCT‐guided breast radiotherapy with Fast‐Forward trial of 26 Gy in five fractions on a Halcyon Linac. This study quantifies Halcyon plan quality, treatment delivery accuracy and efficacy by comparison with those of clinical TrueBeam plans.

**Materials and Methods:**

Ten accelerated partial breast irradiation (APBI) patients (four right, six left) who underwent Fast‐Forward trial at our institute on TrueBeam (6MV beam) were re‐planned on Halcyon (6MV‐FFF). Three site‐specific partial coplanar VMAT arcs and an Acuros‐based dose engine were used. For benchmarking, PTV coverage, organs‐at‐risk (OAR) doses, beam‐on time, and quality assurance (QA) results were compared for both plans.

**Results:**

The average PTV was 806 cc. Compared to TrueBeam plans, Halcyon provided highly conformal and homogeneous plans with similar mean PTVD95 (25.72  vs. 25.73 Gy), both global maximum hotspot < 110% (*p* = 0.954) and similar mean GTV dose (27.04  vs. 26.80 Gy, *p* = 0.093). Halcyon provided lower volume of ipsilateral lung receiving 8 Gy (6.34% vs. 8.18%, p = 0.021), similar heart V1.5 Gy (16.75% vs. 16.92%, p = 0.872), V7Gy (0% vs. 0%), mean heart dose (0.96  vs. 0.9 Gy, *p* = 0.228), lower maximum dose to contralateral breast (3.2  vs. 3.6 Gy, *p* = 0.174), and nipple (19.6  vs. 20.1 Gy, *p* = 0.363). Compared to TrueBeam, Halcyon plans provided similar patient‐specific QA pass rates and independent in‐house Monte Carlo second check results of 99.6% vs. 97.9% (3%/2 mm gamma criteria) and 98.6% versus 99.2%, respectively, suggesting similar treatment delivery accuracy. Halcyon provided shorter beam‐on time (1.49  vs. 1.68 min, *p* = 0.036).

**Conclusion:**

Compared to the SBRT‐dedicated TrueBeam, Halcyon VMAT plans provided similar plan quality and treatment delivery accuracy, yet potentially faster treatment via one‐step patient setup and verification with no patient collision issues. Rapid delivery of daily APBI on Fast‐Forward trial on Halcyon with door‐to‐door patient time < 10 min, could reduce intrafraction motion errors, and improve patient comfort and compliance. We have started treating APBI on Halcyon. Clinical follow‐up results are warranted. We recommend Halcyon users consider implementing the protocol to remote and underserved APBI patients in Halcyon‐only clinics.

## 1 INTRODUCTION

The use of accelerated partial breast irradiation (APBI) of 26 Gy in 5‐fraction daily treatments for patients with node‐negative early‐stage breast cancer, in contrast to whole breast radiotherapy of 40.05  or 42.56 Gy in 15 or 16 fractions in 3 weeks or longer, was recently introduced as the phase III randomized Fast‐Forward clinical trial for breast cancer patients.^1^ With this trial, the 26 Gy in 1 week breast treatment scheme subsequently demonstrated equivalent overall survival and local control rates although there were differences in morbidity outcomes at 5 years. The results of this trial caused us to adopt this treatment regimen as an option for patients eligible for APBI. These patients are typically treated on our SBRT‐dedicated TrueBeam Linac.

We have recently installed a single energy gantry‐mounted linear accelerator, the Varian Halcyon ring delivery system (RDS, V2.0), for image‐guided radiation therapy treatments (Varian Medical Systems, Palo Alto, California, USA). Halcyon RDS is equipped with 6MV‐FFF beam (mean energy = 1.3 MeV and depth of maximal dose = 1.3 cm) and is capable of rotating the gantry at a speed of 4 rotations per minute.^2^, ^3^ This jawless Halcyon RDS is equipped with a new double stacked/staggered multi‐leaf collimator (MLC) configuration for the maximum field size of 28 × 28 cm^2^, with the upper and lower MLCs being offset by 5 mm allowing for a projected 5 mm effective MLC width at isocenter and ultra‐low leakage and transmission of < 0.4% compared to 1.5% for the 6MV beam on TrueBeam. With the less rounded double stacked/staggered MLC design, Halcyon provides smaller penumbra with a smaller dosimetric leaf gap (DLG) of 0.1 mm compared to a 6MV SBRT‐dedicated TrueBeam DLG of 1 mm. For daily image‐guided treatment, Halcyon RDS is equipped with a fast 15 to 30‐s kilovoltage iterative cone beam CT (kV‐iCBCT) imaging system that provides high‐quality iCBCT images for patient setup verification.^4^, ^5^ Due to the introduction of the new “one‐step patient setup and verification,” daily setup times are significantly reduced on the Halcyon RDS that will automatically apply couch shifts during a patient's setup, thus eliminating the need to manually apply isocenter shifts for the therapists.

With these advantages of Halcyon RDS in mind, Cozzi et al.^6^ have shown that fast, safe, and effective treatment delivery is possible with Halcyon RDS for conventionally fractionated daily treatment for head and neck, whole breast, and prostate. Moreover, many other researchers have demonstrated the clinical feasibility of using Halcyon RDS for the conventional daily treatment of whole breast patients using either a dynamically flattened beam (DFB) or FFF beam with tangential fields or field‐in‐field approach including bilateral breast.^7^, ^8^, ^9^, ^10^, ^11^, ^12^, ^13^, ^14^ Our study is to complement those previous investigations and is the first to benchmark on the evaluation and clinical implementation of Halcyon RDS for the hypofractionated Fast‐Forward trial for APBI patients. In this technical report, we have evaluated plan quality, treatment delivery efficiency, and accuracy of APBI on Halcyon by benchmarking with high‐quality APBI treatments that were delivered on our SBRT‐dedicated TrueBeam. We believe that this study will be useful resources for other Halcyon users for implementing APBI in their clinic. This study provides the support and potential benefit of using Halcyon for rapid delivery of APBI treatments for the patients enrolled on this new trial. Based on these findings, the APBI on Halcyon has been clinically implemented in our clinic and patient treatments are ongoing.

## 2 MATERIALS AND METHODS

### 2.1 Fast‐Forward trial and patient characteristics

To briefly mention the implementation of APBI Fast‐Forward trial,^1^ the dose limits are summarized in Table 1. Although this protocol allows beam energies higher than 6 MV beams for 3D‐conformal treatments, for all patients, we have used 6 MV beam and VMAT approach on our TrueBeam Linac. Patient setup and verification were done using the CBCT guidance in‐house clinical protocol similar to SBRT treatment. A comprehensive quality assurance (QA) program was implemented for fast, safe, and accurate treatment of APBI patients in our center.

**TABLE 1:** Fast‐Forward trial dose constraints.

PTV coverage	More than 95% of PTV	95% of prescribed dose
	Less than 5% of PTV	105% or more
	Less than 2% of PTV	107% or more
	Global maximum dose to PTV	<110%
Critical organs		
Ipsilateral lung	Receiving 8 Gy	< 15%
Volume of heart	Receiving 1.5 and 7 Gy	< 30% and 5%

*Note*: Prescription was 26 Gy in five fractions in 1 week.

After obtaining the institutional review board approval, ten consecutive APBI patients (four right, six left) who underwent breast treatment following the Fast‐Forward trial using highly conformal and homogeneous plans via 6MV unilateral coplanar partial VMAT arcs on an SBRT‐dedicated TrueBeam Linac for 26 Gy in five fractions daily, were included in this retrospective validation study.

### 2.2 Patient setup and target definition

Patients were immobilized using the standard breast setup on the breast board in the supine position. A non‐contrast planning CT scan with 3 mm slice thickness (SOMATOM.go CT simulator, Malvern, Pennsylvania, USA) was obtained. The 3D CT scan was brought into Eclipse treatment planning system (TPS, Version 15.6; Varian Medical Systems, Palo Alto, California, USA). Visual tumor was contoured as gross tumor volume (GTV). The clinical target volume (CTV) included the GTV plus an approximate 1.5 cm margin extending from 5 mm below the skin surface to the deep fascia encompassing the post‐surgical skin flaps and underlying soft tissues to the deep fascia; both excluded underlying muscle and rib cage. The planning target volume (PTV) includes a typical margin of 5–10 mm around the CTV to accommodate the daily patient setup error, breast swelling, and breathing motion. The mean PTV volume was 806 ± 687 cc (ranged, 289−2060 cc). The relevant organs‐at‐risk (OAR) that were delineated per Fast‐Forward trial included ipsilateral and contralateral lungs, spinal cord, heart, contralateral breast, nipple, esophagus, and skin.

### 2.3 Clinical APBI‐VMAT plans

Highly conformal and homogeneous clinical APBI VMAT plans were generated in Eclipse TPS using three partial unilateral coplanar arcs on an SBRT‐dedicated TrueBeam Linac (Varian Medical Systems, Palo Alto, California, USA) equipped with a standard Millennium 120 MLC and a 6MV (600 MU/min) beam per our departmental SBRT protocol. The isocenter position was set to the geometric center of the PTV. These partial coplanar arcs had an arc length of approximately 230°, and different collimator angles were manually optimized to reduce MLC tongue‐and‐groove leakage dose throughout the arc rotation. On TrueBeam, the jaw‐tracking option was used during plan optimization to further minimize out‐of‐field dose.^15^ A total dose of 26 Gy in five fractions was prescribed and normalized to ensure at least 95% of the PTV received greater than 95% of the prescribed dose per Fast‐Forward requirement.^1^ No hot spots were allowed outside of the GTV and the global maximum hotspot dose was defined as < 110% (Table 1). All clinical treatment plans were calculated via the advanced AcurosXB (Varian Eclipse TPS, Version 15.6) dose engine for tissue heterogeneity corrections,^16^, ^17^ with 1.25 × 1.25 × 1.25 mm^3^ calculation grid size (CGS), and the Photon Optimizer (PO) MLC algorithm. The dose to medium reporting mode was applied. Planning objectives followed the Fast‐Forward trial's requirements for prescription coverage, target dose heterogeneity, high and low‐dose spillages, and dose‐limiting organ constraints as mentioned above. These patients were treated on TrueBeam every day per CBCT‐guided protocol.

### 2.4 Halcyon APBI‐VMAT plans

For benchmarking, all clinical APBI plans were re‐optimized in Eclipse TPS for Halcyon RDS via the same number of partial arcs, identical collimator rotations, and identical arc geometry (including the TrueBeam VMAT arc length). Additionally, the Halcyon couch structure was inserted, replacing the TrueBeam couch. To meet the Fast‐Forward trial's requirement, optimization objectives were similar, yet tighter to TrueBeam VMAT plans. Identical dose calculation algorithm, dose reporting mode, CGS and PO‐MLC optimizer were used for Halcyon APBI plans as mentioned above. For similar target coverage, Halcyon APBI plans were normalized identically to the corresponding clinical TrueBeam VMAT plans as described above, and the GTV global hot spots were limited to those of the respective TrueBeam APBI plans that were within 110% of the prescription dose.

### 2.5 Plan comparison and data analysis

Following the Fast‐Forward trial's protocol requirement, the clinical TrueBeam and Halcyon APBI plans were compared for target coverage and dose achieved to adjacent OAR. Additionally, delivery efficiency and accuracy were recorded for both platforms. Total number of monitor units (MU) per fraction, beam modulation factor (MF): ratio of total number of MU per fraction to the prescription dose in cGy, and total beam‐on time (BOT) were recorded during portal dosimetry QA measurement at the machine. Dosimetric verification of treatment delivery accuracy on both platforms was performed using a portal dosimetry (PD) VMAT QA procedure,^18^, ^19^ similar to that previously implemented in our clinic for both platforms. At our center, we perform patient‐specific VMAT QA using the electronic portal imaging device (EPID, aS1200 flat panel detector; Varian Medical Systems, Palo Alto, California, USA) mounted on the TrueBeam and Halcyon Linacs, respectively. For PD QA analysis, a gamma evaluation criterion of 3%/2 mm and low dose threshold of 10% were used. The gamma pass rate of greater than 95% was set for each APBI plan. This 400 mm × 400 mm detector with ultra‐high‐resolution of 0.34 mm pixel size makes it an excellent detector for detecting the MLC offset for the VMAT QA. In addition, to verify APBI plans independently, patient‐specific quality assurance was performed using an in‐house Monte Carlo (MC) code for both platforms.^20^ Comparison of dosimetric parameters and plan evaluation metrics was performed using the Wilcoxon Rank Test (nonparametric) or Paired Samples *t*‐test (parametric) and a statistically significance level of *p*‐value of less than 0.05 in SPSS 27 data analysis software (IBM, New York, New York, USA).

## 3 RESULTS

### 3.1 Target coverage and dose to OAR

The target metrics and dose achieved to OAR are displayed in Table 2 for both TrueBeam and Halcyon APBI plans, each demonstrating compliance with the Fast‐Forward protocol requirements. Both platforms produced statistically insignificant differences in target coverage and mean GTV dose, demonstrating similar target dose can be achieved. As per Fast‐Forward protocol requirements, dose achieved to ipsilateral lung and heart were recorded, in addition to other adjacent critical organs (nipple, contralateral breast) for both TrueBeam and Halcyon APBI plans. Both APBI plans met all protocol compliance criteria for OAR sparing and were clinically acceptable for treatment while reviewed by an experienced attending physician. Most comparisons yielded statistically insignificant differences (*p* > 0.05, Table 2) with the exception of an ipsilateral lung receiving 8 Gy on Halcyon was lower by 1.84% (*p* = 0.021), on overage, with a maximum up to 3.82% lower volume of ipsilateral lung irradiated; suggesting potential for better sparing of normal lung on Halcyon RDS for APBI treatments. Although statistically insignificant (*p* = 0.174), the maximal dose to contralateral breast was also systematically lower for all plans on Halcyon compared to TrueBeam APBI treatments.

**TABLE 2:** Summary of plan quality parameters evaluated for all Halcyon plans compared to clinical TrueBeam plans for APBI patients.

Dosimetry parameters	TrueBeam VMAT	Halcyon VMAT	*p*
PTVD95% coverage (Gy)	25.73 ± 0.42 (24.71−26.03)	25.72 ± 0.34 (25.72−26.07)	0.954
Mean dose to GTV (Gy)	26.80 ± 0.17 (26.52−27.08)	27.04 ± 0.34 (26.58−27.74)	0.093
Ipsilateral lung receiving 8 Gy (%)	8.18 ± 4.45 (0.83−14.93)	6.34 ± 3.46 (0.57−11.11)	**0.021**
Mean dose to ipsilateral lung (Gy)	3.53 ± 1.12 (2.13−5.38)	3.37 ± 1.12 (1.95−5.18)	0.419
Volume of heart receiving 1.5 Gy (%)	16.92 ± 10.12 (0.0−30.0)	16.75 ± 9.45 (0.00−29.55)	0.872
Volume of heart receiving 7 Gy (%)	0.0 ± 0.0	0.0 ± 0.0	NA
Mean dose to heart (Gy)	0.90 ± 0.35 (0.33−1.38)	0.96 ± 0.41 (0.36−1.70)	0.228
Contralateral breast maximum dose (Gy)	3.61 ± 1.83 (1.96−8.07)	3.19 ± 1.16 (1.85−5.29)	0.174
Nipple maximum dose (Gy)	20.09 ± 8.88 (0.41−26.54)	19.58 ± 8.21 (0.32−25.77)	0.363

*Note*: Statistically significant values are highlighted in bold.

**FIGURE 1:**
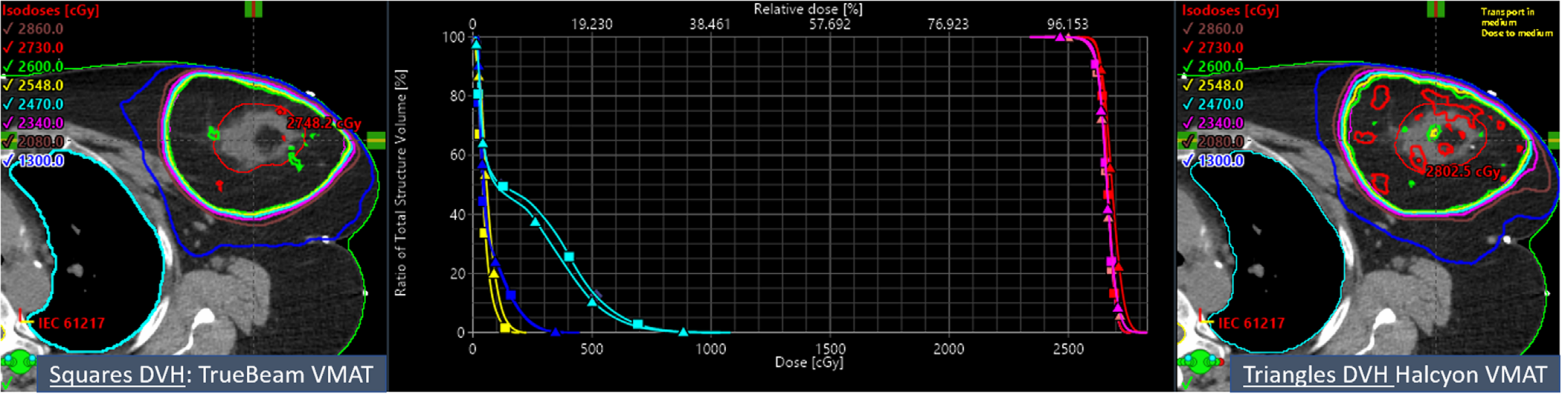
Left: Demonstration of the benchmarked hypofractionated clinical TrueBeam plan (26 Gy in 5‐fraction, daily). Right: the corresponding Halcyon APBI plan for benchmarking the Fast‐Forward trial at our institute. Center: DVH for TrueBeam (squares) and Halcyon (triangles). Red and pink contours are GTV and PTV respectively, achieved similar and highly conformal and homogeneous dose distributions on both platforms. A similar, yet tighter, 50% isodose line (blue) on Halcyon RDS was observed. For similar target coverage, similar contralateral breast dose (yellow) and heart dose (blue) were observed, yet absolute 3% lower ipsilateral lung V8Gy (cyan) was found on Halcyon.

An example patient from the benchmarking cohort representing the typical findings of our validation study is illustrated in Figure 1. The clinical TrueBeam (left panel, squares DVH) versus Halcyon APBI VMAT plan (right panel, triangles DVH) is shown with the corresponding isodose distributions in the axial view, and crosshairs at the isocenter location. Critical organs DVH parameters also shown are ipsilateral lung (cyan), heart (blue), and contralateral breast (yellow) including the DVH comparison for both APBI plans with identical PTV coverage (pink) and similar dose to the GTV (red). Identical target coverage and similar OAR sparing were achieved on both APBI plans with lower dose to ipsilateral lung on Halcyon. In this case, the total MU per fraction, MF, BOT, QA pass rate, and independent MC second physics MU check results were 1025 versus 1326, 1.97 versus 2.55, 1.86  versus 1.66 min, 97.1% versus 99.7%, 1.1% versus 1.7% for clinical TrueBeam and Halcyon APBI plans, respectively (see Figure 1). All dosimetric parameters (including dose to OAR) and QA results were similar between the plans and within the Fast‐Forward trial compliance criteria for APBI treatment.

### 3.2 Treatment delivery accuracy and efficiency

Compared to clinical TrueBeam plans, Halcyon APBI plans delivered higher total MU indicating slightly higher beam modulation. Mean values of total MU (*p* = 0.001) and MF (*p* = 0.001) were 921 and 1.77 for TrueBeam VMAT plans versus 1188 and 2.28 for Halcyon RDS (Table 3), suggesting higher MLC modulation obtained on Halcyon that could have spared the adjacent OAR including the ipsilateral lung volume on Halcyon. The beam‐on time, the PD QA pass rates and independent MC second physics check results for TrueBeam APBI versus Halcyon plans are also shown in Table 3. Despite the higher total MU and relatively larger MF, the mean total beam‐on time at Halcyon APBI plans (1.49 min) was slightly shorter compared to TrueBeam APBI plans (1.77 min) (p = 0.036). That was due to the fact that the maximum achievable dose rate of 800 MU/min, four times faster gantry speed, and two times faster MLC leaf motion on Halcyon contributed to shorter beam‐on time. Moreover, the cumulative overall treatment time could be shorter (< 10 min) because of the previously streamlined “one‐step setup and verification” capability on Halcyon. Faster patient setup and lower beam‐on time will lower the patient's overall time on the table, suggesting a potential reduction in intra‐fraction motion error and patient compliance.

**TABLE 3:** Summary of treatment delivery parameters evaluated for TrueBeam and Halcyon plans for APBI patients.

Treatment and QA Parameters	TrueBeam VMAT	Halcyon VMAT	*p*
Total Monitor Units (MU) per fraction	921 ± 114 (701−1025)	1188 ± 210 (762−1495)	**0.001**
Modulation factor (MF)	1.77 ± 0.22 (1.35−1.97)	2.28 ± 0.40 (1.46−2.87)	**0.001**
Beam‐on time (min)	1.68 ± 0.21 (1.27−1.86)	1.49 ± 0.26 (0.95−1.87)	**0.036**
VMAT QA pass rate (%)	97.85 ± 2.63 (91.5−100)	99.6 ± 0.39 (98.8−100)	0.053
Monte Carlo 2^nd^ check pass rate (%)	99.24 ± 0.43 (98.7−99.9)	98.6 ± 0.52 (97.9−99.5)	**0.001**

*Note*: Statistically significant values are highlighted in bold.

As mentioned earlier, treatment delivery accuracy of APBI treatments was evaluated by delivering each QA plan on both platforms via on‐board EPID and recording the clinical gamma pass rates via portal dosimetry. While, the better TPS model could deliver the higher VMAT QA pass rates, the dose delivery accuracy of our clinical TrueBeam APBI plans and the corresponding Halcyon plans were 97.9% and 99.6% on average, respectively, using 3%/2 mm clinical gamma criteria. Although, statistically significant (*p* = 0.001), both platforms showed high accuracy (average agreement within 1.5% compared to the corresponding original Eclipse plans) for independent dose verification via an in‐house MC program for second physics check.

### 3.3 First APBI treatment on Halcyon

#### 3.3.1 Plan quality, treatment delivery accuracy, and efficiency

**FIGURE 2:**
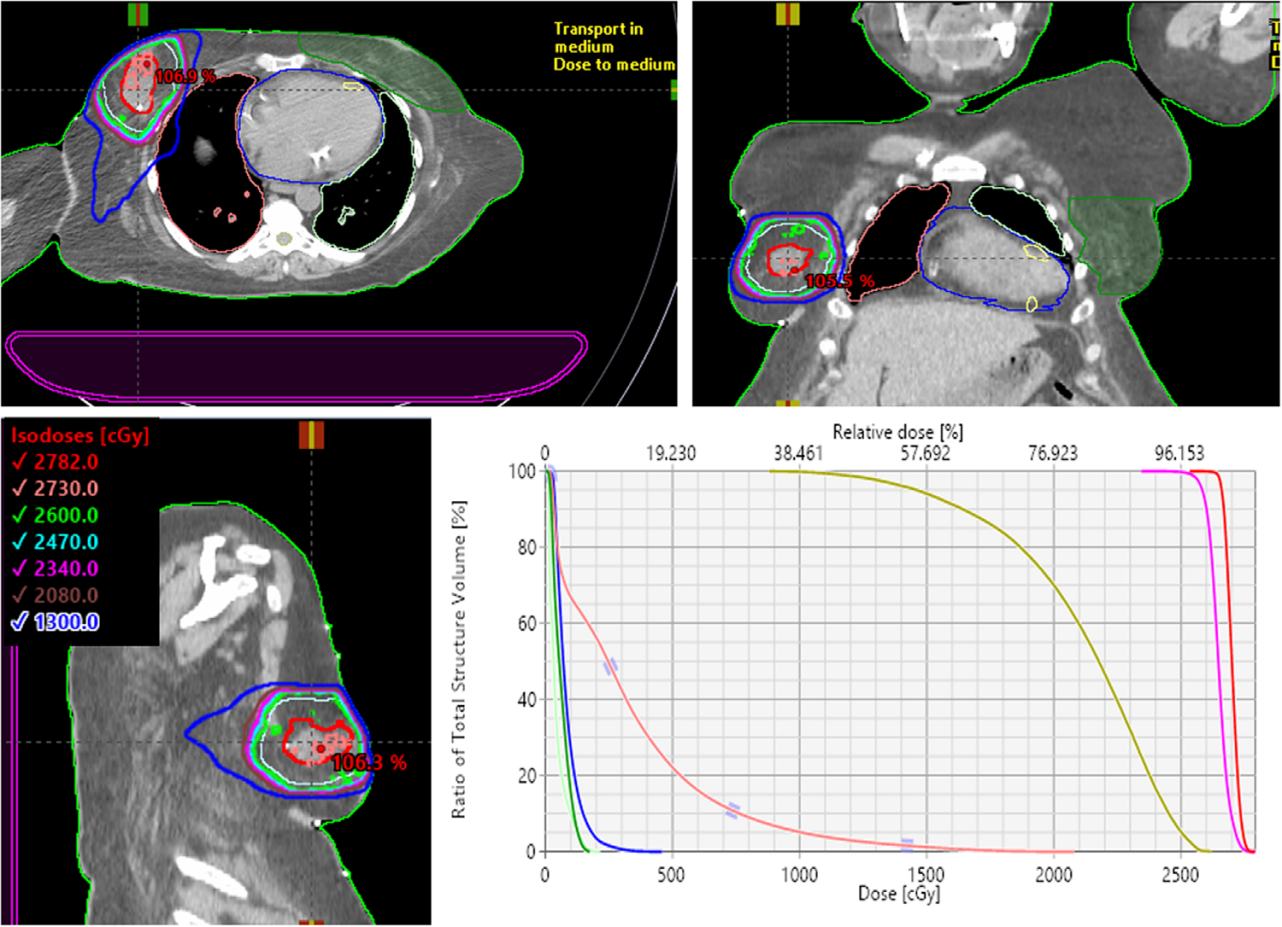
Demonstration of the Halcyon APBI‐VMAT plan for the right breast patient, the first treated with the Fast‐Forward trial (26 Gy in 5‐fraction, daily) in our center. The isodose lines and the DVH parameters are shown. The crosshairs show the isocenter location. Critical structures shown were heart (blue), ipsilateral (light pink) and contralateral (light green) lung, contralateral breast (green) and nipple (dark yellow). The GTV (red) and PTV (pink) received highly conformal and homogeneous dose while selectively sparing all the critical organs including the use of sector avoidance for the right arm.

Based on these promising validation results, we have fully implemented APBI treatment in our Halcyon RDS. The first patient who underwent APBI on our Halcyon received a total of 26 Gy in five treatments every day for a right breast cancer (Figure 2). The PTV size was 233.2 cc (7.6 cm diameter). Three coplanar partial arcs with an arc length of 230^o^ were used with three different collimator settings. In this case, 95% of the PTV received 99.4% (25.85 Gy) of the prescription dose with GTV mean dose of 27 Gy, hot spot within 107% inside the GTV, all parameters were compliant with the Fast‐Forward trial. It was observed that: ipsilateral lung V8Gy (< 9%) and V10Gy (< 5%); heart V1.5 (< 9.6%) and V7 (0%); maximum dose to contralateral breast (< 1.74 Gy); and maximum dose to nipple (< 24.91 Gy) were all well below the protocol's requirement. The total MU per fraction was 1325. The MF and total beam‐on time were 2.55 and 1.66 min, respectively. The net treatment time (from first arc on until last arc off, including second and third arc preparation time) was about 2.12 min. For this patient, recorded mean couch time (including one‐step patient setup, 30‐s kV conebeam CT imaging, and tumor matching) was less than 10 min–the patient's door‐to‐door time.

Figure 2 shows the details of APBI dose distributions in three views through the isocenter for this patient treated with Halcyon VMAT plan including the Halcyon couch insert. This patient was set up on the standard breast board with no collision concern. Halcyon RDS provided a clinically desirable tighter 50% isodose line (blue), also sparing the right arm via sector avoidances, as needed.

**FIGURE 3:**
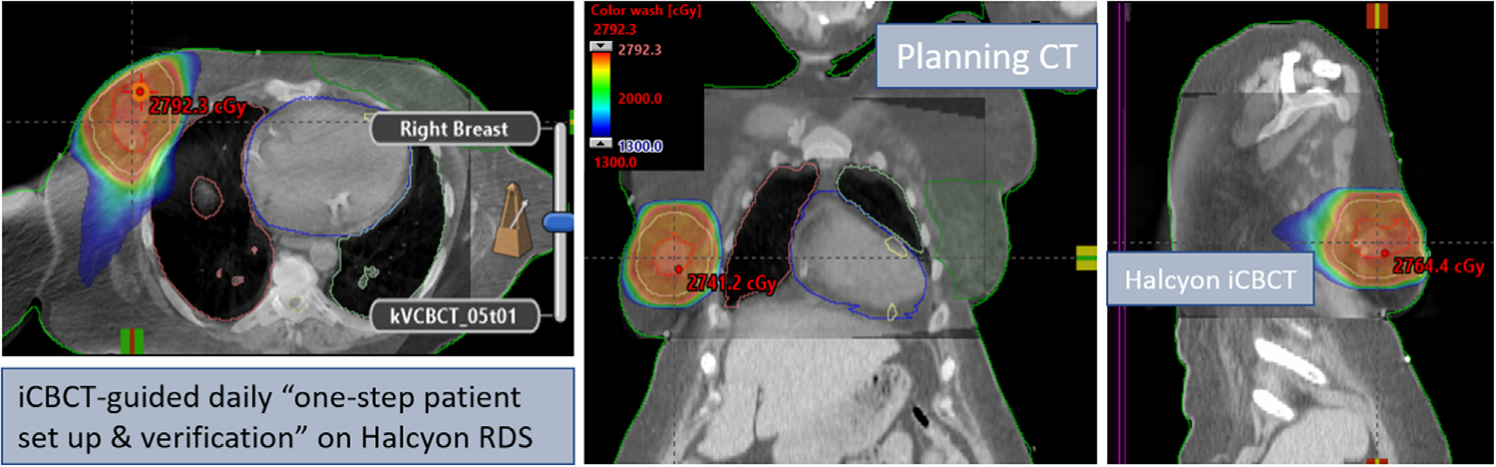
Axial, coronal, and sagittal views of Halcyon kV‐iCBCT images (see inset) co‐registered with planning CT images (see back of coronal and sagittal views) used for image‐guided APBI treatment on Halcyon. In addition to anatomical landmarks, the planned dose cloud was superimposed for this treatment. Halcyon kV‐iCBCT images were acquired in the treatment position on breast board. The 3D soft‐tissue matching was performed via auto‐registration of online kV‐iCBCT scan with the planning CT followed by manually fine‐tuning for more accurate patient setup corrections.

Pre‐treatment PD VMAT QA pass rate was 99.2% with a 3%/2 mm gamma passing criteria. The net treatment time (from first beam‐on until last beam off, including second and third beam preparation) was about 2 min. Recorded overall door‐to‐door time for this patient on Halcyon was < 10 min. This patient was initially positioned using external body marks and in‐room blue lasers, followed by the automated one‐step patient setup via shifts from Halcyon's blue lasers to the yellow lasers and a 30‐s pre‐treatment free‐breathing kV‐iCBCT scan. An in‐house IGRT/SBRT clinical protocol was applied to co‐register the pre‐treatment kV‐iCBCT images with the planning CT scan (see Figure 3). Image registration was performed automatically based on region of interest and anatomical landmarks. Soft‐tissue registration was followed by manual refinement and confirmed by the treating physician and physicist. The patient position was then corrected for three degrees of freedom (3DOF) according to the results of soft tissue registration, and the treatment was delivered.

Those 3DOF couch corrections were within the limits of departmental APBI IGRT protocol requirements for this patient (translational shifts within ± 3.0 mm in each direction). Figure 3 shows the planned isodose colorwash superimposed with the daily Halcyon kV‐iCBCT images after the translational couch corrections were applied for this fraction. The entire imaging and treatment delivery sequences were monitored at the treatment console and verified by the treating physician.

## 4 DISCUSSION

In this technical report, we have demonstrated our initial clinical experiences of benchmarking Halcyon RDS for fast, safe, and accurate CBCT‐guided treatment of APBI patients who underwent the Fast‐Forward trial of 26 Gy in five treatments, daily. Our Halcyon VMAT plans via three partial unilateral arcs with stacked/staggered MLCs and manually selected patient specific collimator angles to minimize leakage dose from MLC travelling surpassed the clinical TrueBeam plans. Halcyon APBI plans were highly conformal, homogeneous, achieved adequate target coverage, and effectively spared the adjacent critical organs (Table 2) compared to the SBRT‐dedicated TrueBeam plans following the Fast‐Forward trial's requirement. For all patients, the Halcyon APBI plans provided similar or better plan quality with slightly lower maximum dose to ipsilateral lung (Table 2). The overall door‐to‐door patient time was less than 10 min per fraction with no patient collision concern. After all the promising benchmarking results, for the first patient, the independent MC second physics check agreed within 1.5%, and the patient‐specific pre‐treatment VMAT QA gamma passing rates of 99.1% (for 3%/2 mm clinical gamma passing criteria) demonstrated an excellent potential for fast, reliable, and accurate delivery of the APBI treatment on Halcyon RDS. Due to the fully automated one‐step patient setup, verification, and treatment delivery workflow, we have clinically implemented Halcyon for our APBI treatments.

Achieving acceptable dose limits in regards to PTV coverage constraints and normal tissue organ constraints is of high priority. As seen in the Fast‐Forward trial, there was a significant increase in morbidity when comparing 26 Gy in five fractions with 27 Gy in five fractions. This shows a narrow therapeutic index in order to both provide adequate local control and acceptable morbidity. Therefore, the ability of Halcyon to meet the dosimetric PTV criteria of Fast‐Forward and still provide a lower ipsilateral lung dose shows that the five‐fraction regimen can be safely used on Halcyon RDS.

A few things should be mentioned regarding Halcyon APBI. In addition to providing rapid APBI treatments in community centers, Halcyon RDS could potentially support academic centers with high‐volume SBRT clinics in the expansion of their SBRT program(s) or transfer of APBI patients to Halcyon for similar yet faster treatment, if needed. Moreover, in the case of a long machine (C‐arm Linac) downtime, quickly replanning APBI treatment on Halcyon would be very helpful for not delaying the treatment course for patients undergoing the Fast‐Forward trial. However, we mention a few caveats in this study. One caveat is a TrueBeam Linac with a 6DOF couch may better reproduce rotational patient setup errors in treatment delivery. However, as of now, the exact dosimetric impacts of 6DOF versus 3DOF couch corrections for a large target volume such as in APBI treatments on Halcyon are not known and it is an interest of our future investigation. Another caveat is that the current Halcyon RDS does not yet have a full package of motion management system available and clinically implemented. Thus, as of now, fully automated deep inspiration breath‐hold (DIBH) for APBI treatments is not available on our Halcyon.

In summary, rapid delivery of iCBCT‐guided APBI treatments (< 10 min) is possible with the use of Halcyon RDS, potentially benefiting patients by reducing intrafraction motion error, and improving patient comfort and compliance with no patient collision issues. To provide them a faster, safer, and overall improved quality of radiation treatment, at the time of this report, we are actively recruiting APBI patients on the Fast‐Forward trial's dosing scheme on Halcyon RDS. Our ongoing study includes the automation of a RapidPlan knowledge‐based planning (KBP) model for generating high‐quality APBI plans for breast cancer patients on Halcyon RDS, similar to the one recently developed by Costa et al.^21^ Moreover, clinical follow‐up results of these APBI patients who underwent the Fast‐Forward trial on Halcyon is warranted.

## 5 CONCLUSION

Compared to the SBRT‐dedicated TrueBeam, Halcyon APBI plans provided similar plan quality and treatment delivery accuracy, yet potentially faster treatment via a fully‐automated one‐step patient setup and verification procedure with no patient collision concern. Rapid delivery of daily APBI on the Fast‐Forward trial on Halcyon with door‐to‐door patient time < 10 min, reduced intrafraction motion errors, and improved patient comfort and compliance were achieved. We have started treating APBI via the Fast‐Forward trial on Halcyon RDS. We recommend Halcyon users considering implementing the Fast‐Forward protocol to provide the service of high‐quality therapeutic breast treatments to remote and underserved APBI patients in Halcyon‐only clinics.

## AUTHOR CONTRIBUTIONS

Damodar Pokhrel and Mark E. Bernard conceived the project. Damodar Pokhrel generated the treatment plans. Mason Smith, Alexander Volk, and Damodar Pokhrel collected and analyzed the data. Mark E. Bernard provided his clinical expertise and supervision of the paper. Damodar Pokhrel drafted the preliminary manuscript and all co‐authors revised and approved the final manuscript.

## CONFLICT OF INTEREST STATEMENT

The authors report no conflicts of interest.

## Data Availability

Research data are not shared.
